# Steering and Encoding the Polarization of the Second Harmonic in the Visible with a Monolithic LiNbO_3_ Metasurface

**DOI:** 10.1021/acsphotonics.1c00026

**Published:** 2021-02-19

**Authors:** Luca Carletti, Attilio Zilli, Fabio Moia, Andrea Toma, Marco Finazzi, Costantino De Angelis, Dragomir N. Neshev, Michele Celebrano

**Affiliations:** † Department of Information Engineering, University of Brescia, Via Branze 38, 25123 Brescia, Italy; ‡ Physics Department, Politecnico di Milano, Piazza Leonardo da Vinci 32, 20133 Milano, Italy; ¶ Istituto Italiano di Tecnologia, Via Morego 30, 16163 Genova, Italy; § ARC Centre of Excellence for Transformative Meta-Optical Systems (TMOS), Research School of Physics, Australian National University, 58 Mills Road, Acton, ACT 2601, Australia

**Keywords:** nonlinear nanophotonics, nonlinear diffraction, lithium niobate, metasurface, second-harmonic generation

## Abstract

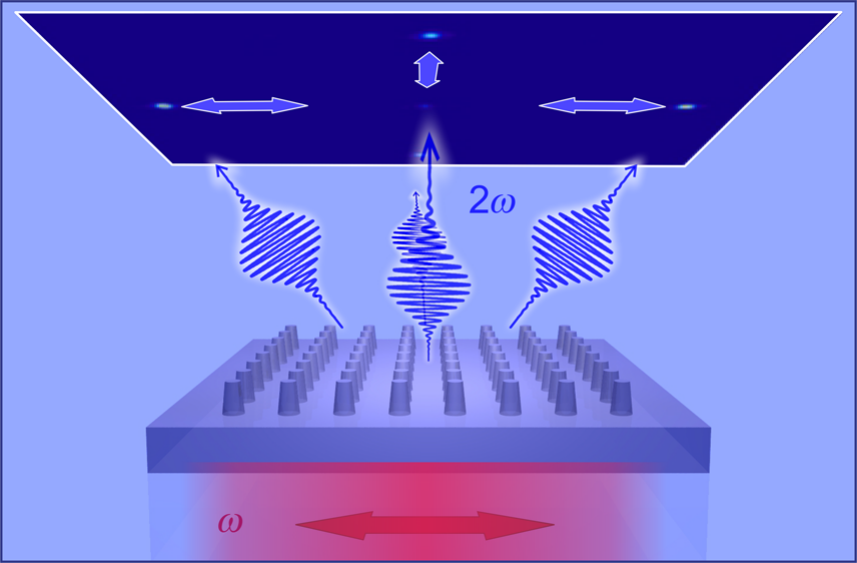

Nonlinear metasurfaces constitute a key asset in meta-optics, given their ability to scale down nonlinear optics to sub-micrometer thicknesses. To date, nonlinear metasurfaces have been mainly realized using narrow band gap semiconductors, with operation limited to the near-infrared range. Nonlinear meta-optics in the visible range can be realized using transparent materials with high refractive index, such as lithium niobate (LiNbO_3_). Yet, efficient operation in this strategic spectral window has been so far prevented by the nanofabrication challenges associated with LiNbO_3_, which considerably limit the aspect ratio and minimum size of the nanostructures (i.e., meta-atoms). Here we demonstrate the first monolithic nonlinear periodic metasurface based on LiNbO_3_ and operating in the visible range. Realized through ion beam milling, our metasurface features a second-harmonic (SH) conversion efficiency of 2.40 × 10^–8^ at a pump intensity as low as 0.5 GW/cm^2^. By tuning the pump polarization, we demonstrate efficient steering and polarization encoding into narrow SH diffraction orders, opening novel opportunities for polarization-encoded nonlinear meta-optics.

The interest in nonlinear nanophotonics based on dielectric materials has been rising fast during the past decade.^1−3^ The employment of materials with high refractive index and large nonlinear susceptibility along with low absorption in the near-infrared (NIR) fostered the enhancement of nonlinear effects at the nanoscale to unprecedented levels in this wavelength range.^4−10^ Metasurfaces composed by ensembles of dielectric nanoresonators with subwavelength dimensions, i.e., meta-atoms, have been employed to enhance^11,12^ and control^10,13−15^ nonlinearly generated light, providing new tools for ultracompact nonlinear meta-optics. Thus far, nonlinear metasurfaces have been applied to the generation of either third-harmonic (TH) or second-harmonic (SH), depending on the meta-atom material composition. Group IV semiconductors have been used for third-harmonic generation (THG),^12,14,15^ whereas III–V semiconducting compounds and alloys have been used for second-harmonic generation (SHG).^11,13,16^ The choice of these materials is mainly driven by the availability of state-of-the-art nanofabrication technologies for CMOS-compatible platforms.^1^ Nonetheless, most semiconductors feature narrow band gaps, with onsets of the optical absorption in the NIR wavelength range.^6,7,13^ This has hindered thus far the development of low-loss nonlinear meta-optics at visible wavelengths.

Lithium niobate (LiNbO_3_) is a material with a wide transparency window spanning from the ultraviolet to the mid-infrared, which enables operation in the visible (VIS) spectral range. LiNbO_3_ features a moderately high refractive index and a sizable second-order nonlinear optical response. These unique properties, which make LiNbO_3_ one of the most widely employed materials in nonlinear photonics and electro-optics, motivated the recent efforts toward the realization of LiNbO_3_-based nanostructures^17−19^ and metasurfaces.^20−25^ Yet, the realization of monolithic LiNbO_3_ metasurfaces has been hindered by the complexity of the nanofabrication processes. In particular, electron-beam lithography (EBL) combined with Ar^+^-based reactive ion etching—a commonly employed technique to realize LiNbO_3_-based integrated photonics circuits^26,27^—limits the realization of nanostructures with a high aspect ratio, which is crucial to enhance field confinement and cavity effects in the VIS range. Focused ion beam (FIB) milling, on the other hand, allows fabricating nanostructures with high aspect ratio and steep side walls, which is essential to deploy the full potential of LiNbO_3_-based nanostructures and metasurfaces. This approach has been recently applied to realize LiNbO_3_-based metasurfaces limited to a 1D periodicity.^20,23^

In this work, we realize a monolithic LiNbO_3_ nonlinear metasurface from a *z*-cut LiNbO_3_ thin film and characterize its ability to up-convert NIR light to the VIS range via SHG. Periodic metasurfaces, such as the one reported here, provide multiple degrees of freedom for engineering the properties of the emitted nonlinear light. In particular, (i) the lattice periodicity allows steering the emitted light into narrow diffraction orders, (ii) the geometry of the individual meta-atom along with its crystalline orientation and lattice structure enables one to control the polarization state of the emitted light based on that of the excitation light.^13^ In addition, (iii) the design of the meta-atoms governs the local amplitude and phase of the emitted light, which can be employed to shape the wavefront of the nonlinear emitted light.^10,14,15,28,29^ In our metasurface the SHG process is driven by a magnetic dipole (MD) resonance at the fundamental wavelength (FW) in each individual meta-atom, which efficiently couples to the in-plane field components of a linearly polarized beam impinging at normal incidence. The energy transfer to the SH is promoted by the out-of-plane component of the nonlinear polarization, while the interference between higher order multipoles in the meta-atom at the SH wavelength along with the lattice periodicity enables the efficient emission of the SH into diffraction orders (see Figure 1a). This mechanism also allows to encode the polarization of the pump beam into specific SH polarization states and specific diffraction orders. Our experimental results are in excellent agreement with the numerical simulations we employed to design the metasurfaces,^22^ providing a full description of both the linear and nonlinear optical processes at work.

**Figure 1:**
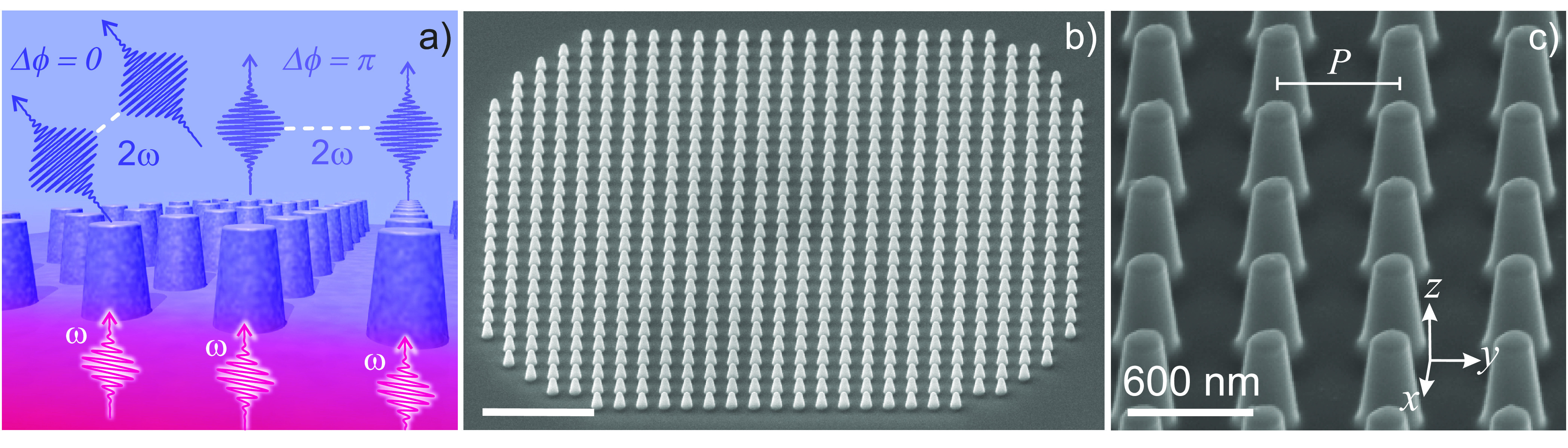
LiNbO_3_ metasurface operating principle and geometry. (a) Sketch of the diffraction mechanism at play in the metasurfaces. The pump at angular frequency ω impinges on the LiNbO_3_ nanopillar grating from the substrate side. The SH, generated at angular frequency 2ω, is removed from the zeroth diffraction order and directed to the first diffraction orders thanks to the interference between the emission patterns of the individual nanopillars. (b) Electron microscopy image of one of the realized metasurfaces. Scale bar is 3 μm. (c) Zoom of the nanopillars showing a ∼80° side-wall inclination and a flat top obtained at the end of the process. The base radius of each nanopillar is 175 nm, the height is 420 nm, and the array pitch, *P*, is 590 nm. The metasurface lies in the *xy* Cartesian plane, with the extraordinary axis of LiNbO_3_ along *z*.

The samples are realized on commercially available *z*-cut LiNbO_3_ films grown on a transparent quartz substrate (NanoLN, Jinan Jingzheng Electronics Co.). The metasurfaces are obtained by directly milling the 5-μm thick LiNbO_3_ film by FIB (FEI, dual-beam Helios Nanolab 650). A detailed description of the fabrication process is presented in Section S.II of the supporting information (SI). Briefly, Ga^+^ ions are emitted with a current of 230 pA and accelerated by a voltage of 30 keV. The overall ion dose is optimized to achieve a patterning depth of around 420 nm. To avoid charging effects, a 200 nm thick Cr layer is deposited by radio frequency sputtering before the ion milling and then removed in standard chrome etch solution (Micro Resist Technology GmbH). The Cr film acts also as a sacrificial layer to prevent further alterations induced by Ga^+^ ions on the top surface of the LiNbO_3_ pillars during the milling process. Figure 1b shows a scanning electron microscopy image of the realized metasurface, which spans an area of about 15 μm × 15 μm with an array pitch, *P*, of 590 nm, and a nanopillar radius, *R*, of 175 nm (Figure 1b and c). To design such optimized geometry, similarly to ref (22), we employed full-vectorial numerical simulations that are described in the SI. The FIB technique allowed attaining an angle of the side walls of the nanopillars of 83.6°; see Figure 1c. It is worth noting that, since the pump beam propagates along the extraordinary axis (*z*-cut), the SHG process is highly inefficient in the LiNbO_3_ film.^22^ Therefore, the SHG enhancement is mainly driven by the optical resonances of the metasurface.

Figure 2a shows the Cartesian multipolar decomposition of the resonances that underpin the optical response of the optimized metasurface.^30^ The marked MD resonance around 830 nm is responsible for the enhanced light–matter interaction in the nanopillars at the FW. Concurrently, the magnetic quadrupole (MQ) and electric dipole (ED) resonances in the structure around 415 nm contribute to the efficient conversion of the impinging light into the SH and its re-emission to the far field. Considering the *z*-cut wafer employed to realize the metasurface, the largest component of the induced nonlinear polarization density, **P**^SH^, is *P*_*z*_^SH^ since *d*_33_ is about 1 order of magnitude larger than the other nonlinear tensor elements (see section S.V of the SI). Therefore, *E*_*z*_ is expected to be the most relevant field component, both at the FW and at the SH. The *E*_*z*_ intensity enhancement in the nanopillar, shown in Figure 2b, features two peaks that overlap with either the FW or the SH. In particular, the peak at the FW stems from the MD resonance, confirming its key role in the SHG enhancement. Conversely, the peak at SH wavelength features the superposition between an ED and an MQ resonance, which plays a key role in the polarized emission of the SH (see below and the SI). The corresponding field intensity distributions inside the meta-atom at the FW and SH are shown in Figure 2c and d, respectively. Concurrently, the calculated SH field distribution (see Figure 2e) indicates a favorable SH emission toward higher collection angles, which can be efficiently overlapped in *k*-space to the first-order diffraction of the metasurface. Before assessing the nonlinear performances of the optimized metasurface, we compared the simulated linear transmittance of the structure with the measured one. Figure 3a shows the simulated transmittance spectra in the VIS–NIR range for three metasurfaces with *P* = 590 nm and varying *R*. In the NIR, all spectra exhibit a transmittance increasing with the wavelength corresponding with the MD resonance (see Figure 2a). By comparing the transmittance of the optimized metasurface (*R* = 175 nm) against the other structures (*R* = 150 and 200 nm), one can verify that larger radii lead to a broadening of the extinction range and to a red-shift toward the NIR. Figure 3b shows the measured transmittance spectra of the metasurfaces simulated in Figure 3a. The spectra were recorded by focusing an incoherent white light from a tungsten lamp on the metasurfaces and coupling the transmitted light to a VIS–NIR spectrometer (Andor Shamrock 303 + iKON-M934 CCD Camera, Oxford Instruments). The light transmitted by the unpatterned LiNbO_3_ substrate was used as reference. The agreement between simulated (Figure 3a) and measured (Figure 3b) spectra demonstrate the quality of the nanofabrication process.

**Figure 2:**
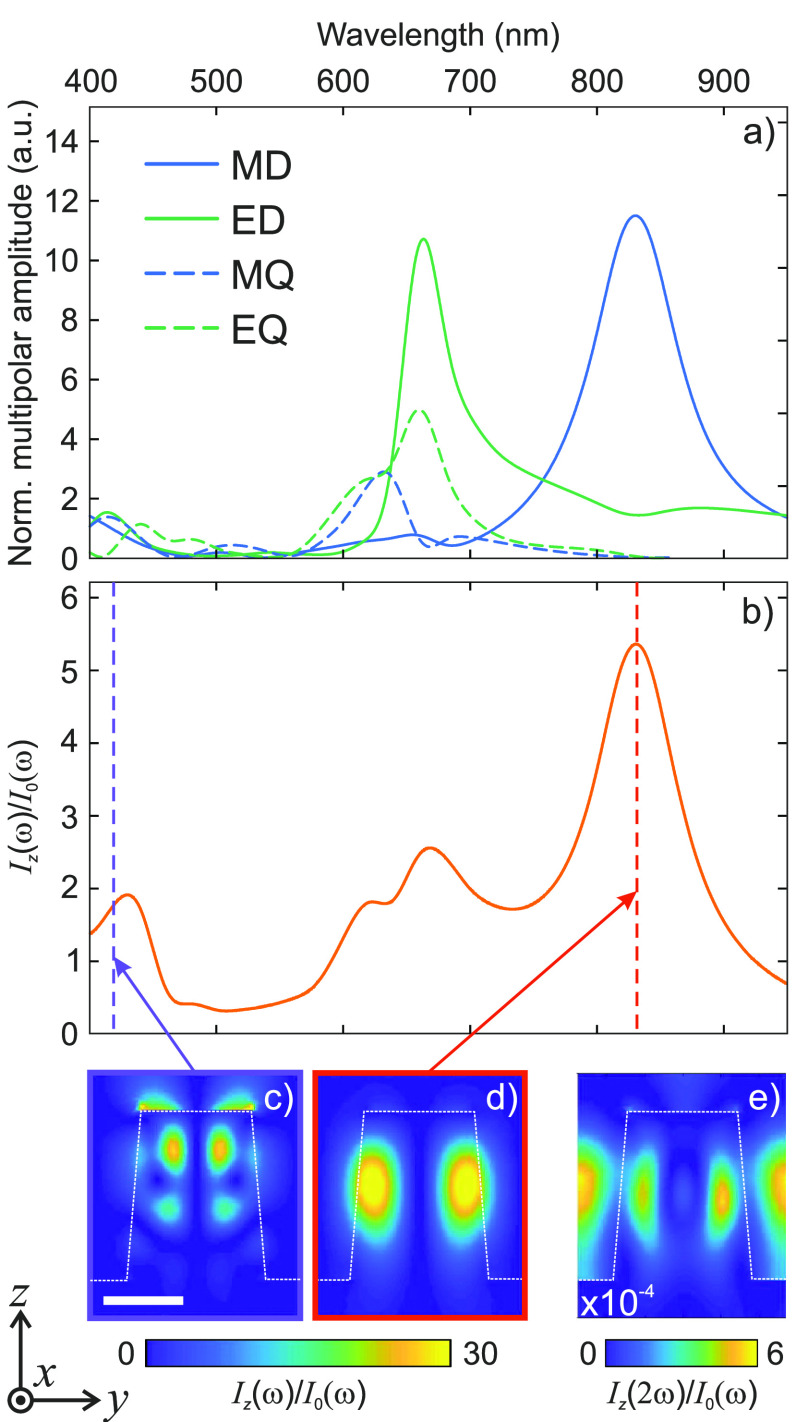
Multipolar decomposition. (a) The blue lines represent the scattering contribution of the magnetic dipole (MD, solid) and of the magnetic quadrupole (MQ, dashed) modes, while the green lines represent that of the electric dipole (ED, solid) and of the electric quadrupole (EQ, dashed) modes. (b) Intensity enhancement averaged in the meta-atom volume. The metasurface parameters are *R* = 175 nm and *P* = 590 nm. (c, d) Intensity enhancement of the *z*-component of the electric field in the meta-atom at 415 nm (c, violet frame) and 830 nm (d, red frame). The violet and red dashed vertical lines in (b) indicate the wavelength corresponding to the maps (c) and (d), respectively. (e) Simulated SH intensity normalized to the incident one.

**Figure 3:**
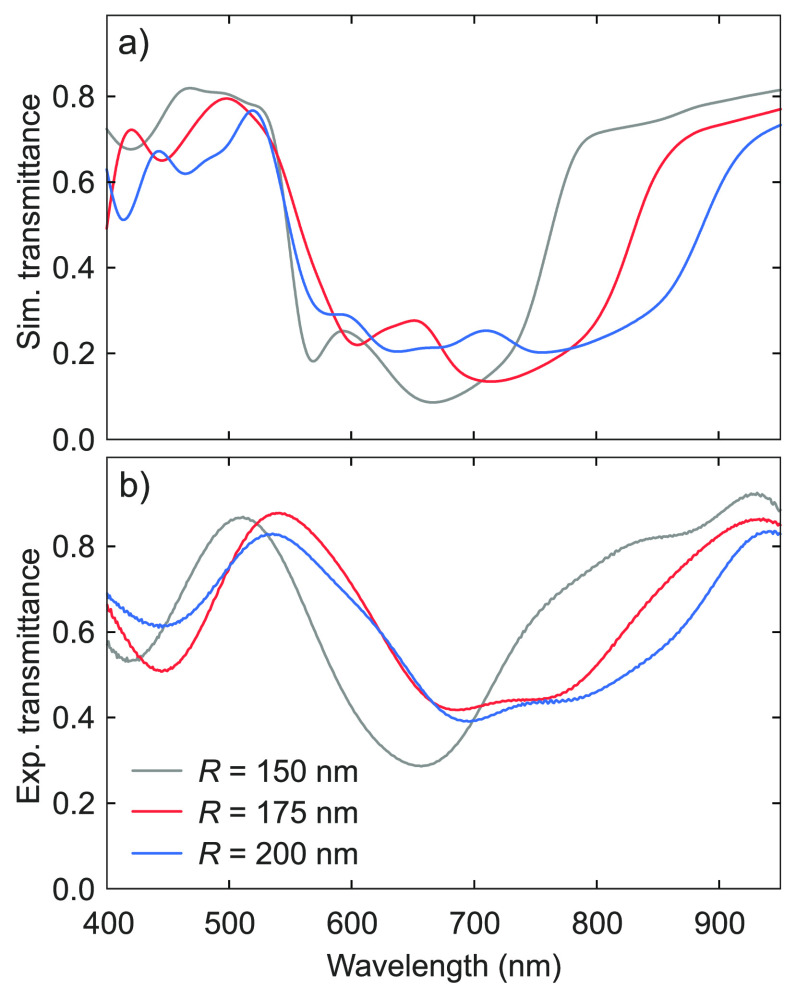
Linear transmittance. (a) Simulated transmittance spectra of LiNbO_3_ metasurfaces as a function of the meta-atom radius *R* as indicated by the shared legend in panel (c). (b) Measured transmittance spectra of the metasurfaces simulated in (a).

To characterize the SH emission properties of the metasurface, we employed a home-built nonlinear inverted microscope described in detail in Section S.I of the SI. Briefly, the sample was mounted with the metasurface facing the objective, while the excitation came from the LiNbO_3_ substrate side, by loosely focusing the beam with a 60 mm focal length lens. This produced a 15-μm wide (full width at half-maximum, FWHM) excitation spot, matching the lateral size of the metasurface. We employed excitation average powers up to 10 mW (∼0.5 GW/cm^2^ peak intensity), delivered by a tunable (680 to 1080 nm) Ti-sapphire laser (Chameleon Ultra II, Coherent Inc.), yielding 140 fs pulses at 80 MHz repetition rate. The emitted SH radiation was collected using a 0.85 numerical aperture (NA) objective, which corresponds to a maximum collection angle of about 58°, and the back focal plane (BFP) of the objective was imaged by a CCD camera (see details in the SI). As the metasurface periodicity is larger than the SH wavelength, the first diffraction orders are expected at
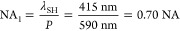
1corresponding to an angle of 44.7°. Figure 4a,b show exemplary BFP SHG images recorded using horizontal and vertical input polarization, respectively. Narrow emission spots (see bottom-left insets) appear at 0.70 NA as expected. In particular, the diffraction orders co-polarized with the FW beam are about 4 times more intense than the cross-polarized ones, which is in good agreement with the simulations (see insets in Figure 4c,d). The BFP images recorded on the unpatterned LiNbO_3_, shown in Figure 4c and d, highlight that the intensity of the (0,0) diffraction order is insensitive to the polarization of the excitation beam. By comparing the BFP SHG images acquired on the metasurface with those on the unpatterned LiNbO_3_ substrate one can readily notice a 1 order of magnitude suppression of the (0,0) diffraction order in the metasurface. This efficient diffraction is ascribed to the choice of *z*-cut LiNbO_3_ substrate for the realization of our nonlinear metasurfaces.

**Figure 4:**
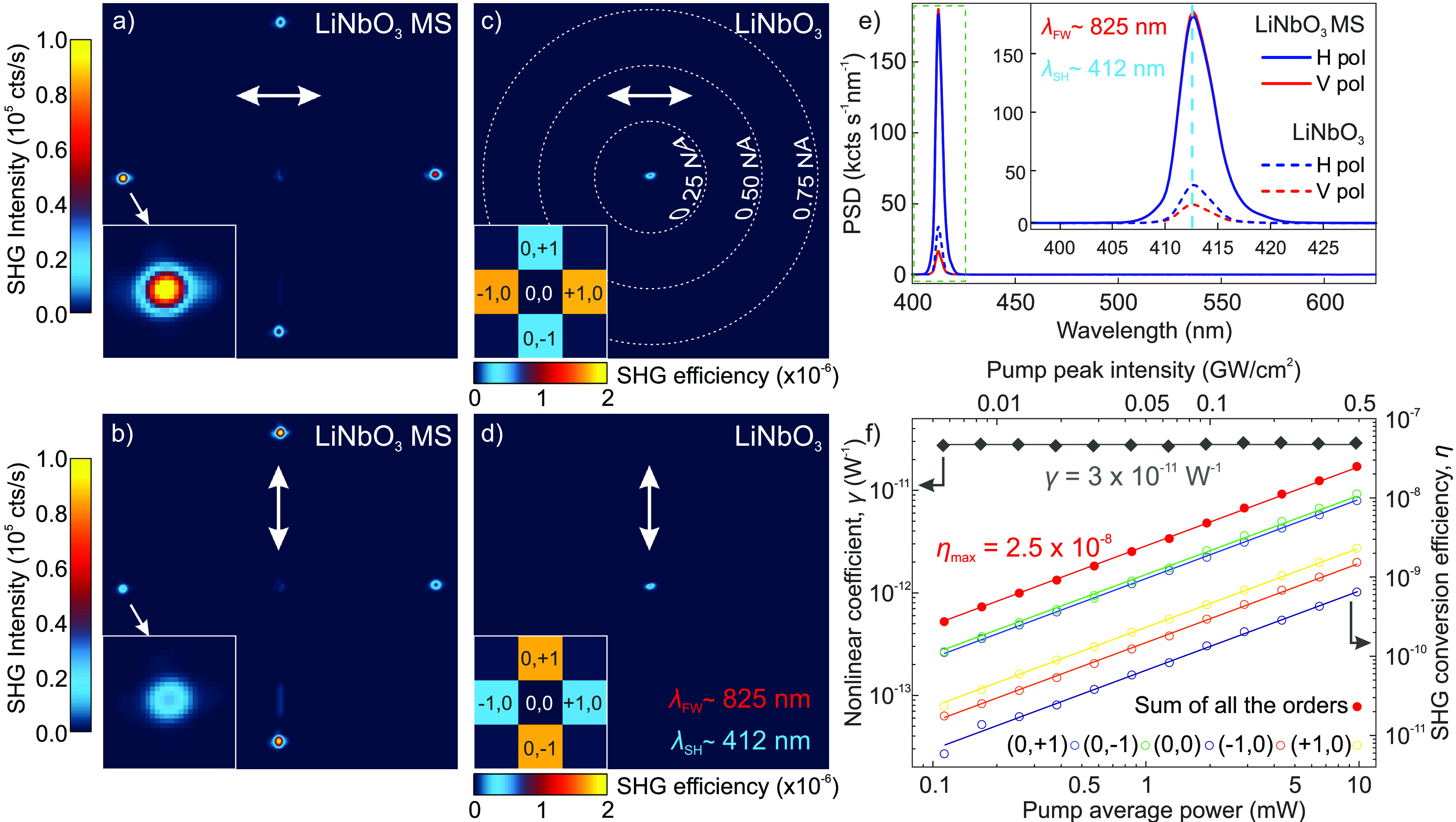
Nonlinear emission from the LiNbO_3_ metasurface. (a) BFP image of the SH emission from the optimized metasurface (MS) (*R* = 175 nm, *P* = 590 nm) excited with horizontal polarization. Inset: zoom of the (−1,0) diffraction order. (b) BFP image of the same metasurface illuminated with vertical polarization. Inset: zoom of the (−1,0) diffraction order. (c, d) BFP images acquired on the unpatterned LiNbO_3_ using either horizontal (c) or (d) vertical excitation polarization. The FW employed to acquire the BFP maps and the corresponding SH are ∼825 and ∼412 nm, respectively. The insets of (c) and (d) are the simulated SHG intensities emitted by the metasurfaces in each diffraction order, for comparison with (a) and (b), respectively. (e) (Solid lines) spectrum of the nonlinear emission by the metasurface under horizontal (blue) and vertical (red) exciting polarization, compared to the nonlinear emission by the unpatterned LiNbO_3_ substrate (dashed lines). The input pulse peak intensity is 0.5 GW/cm^2^. (f) SHG conversion efficiency of the diffraction orders as a function of the excitation intensity for vertical pump polarization as in (b). The straight colored lines interpolating the experimental data are linear fits with slope 1.

The BFP SHG maps were acquired by chromatically filtering the transmitted FW; see section S.I of the SI. In fact, the collected emission spectra in Figure 4e, which show the presence of the sole SH peak centered at 412.5 nm when an FW of 825 nm is employed. To further verify the SH nature of the signal, we acquired power-dependent curves for each individual diffraction order. Figure 4f shows the SH conversion efficiency, η ≡ *P*_av_^SH^/*P*_av_^FW^, as a function of the excitation power and peak intensity, where *P*_av_^SH^ indicates the SH power emitted—once the detection efficiency of the experimental setup is accounted for (see the SI for details)—and *P*_av_^FW^ is the excitation power. As expected, the dependence of η on the excitation power or peak intensity is linear, given that the system operates in the undepleted pump regime. The power curves in Figure 4f together with the emission spectra in Figure 4e confirm that the emission from the metasurface is pure SHG. Comparing the overall emission from the metasurface with that from the unpatterned LiNbO_3_ film (see Figure 4e), one can note an order of magnitude emission enhancement by the metasurface. Even employing pump peak intensities below the GW/cm^2^ (∼0.5 GW/cm^2^), we obtained an SH emitted power *P_av_^SH^* ≈ 0.25 nW from the metasurface, corresponding to η ≈ 2.50 × 10^–8^ (see red dots Figure 4f). We also extrapolated the nonlinear coefficient γ ≡ *P*_pk_^SH^/(*P*_pk_^FW^)^2^ ≈ 3 × 10^–11^ W^–1^ that, being a function of the SH (*P*_pk_^SH^) and FW (*P*_pk_^FW^) peak powers, allows assessing the nonlinear performances of the sample independently of the excitation source (i.e., pulse width and repetition rate). The nonlinear performances of the metasurface were also assessed via simulations using the approach described in the SI and in ref (22) and returned η ≈ 5 × 10^–6^ and γ ≈ 5.8 × 10^–9^ W^–1^. These values are 1 order of magnitude higher than those numerically obtained from the bare LiNbO_3_ film following the same approach, which is in excellent agreement with the enhancement found in the experiment. The 2 orders of magnitude difference between the measured and simulated conversion efficiencies can be ascribed to uncertainties in the estimation of the input intensity and of the optical transmittance of the detection path as well as to quantitative deviations of the numerical simulations from the experiments. For instance, while in the experiment excitation is provided by weakly focused ultrashort pulses (∼15 nm bandwidth), the simulations are performed using a monochromatic plane wave. It is also worth noting that the value of γ determined experimentally is in line with that of a recently reported LiNbO_3_-based metasurface operating in the NIR.^24^

To assess the wavelength-dependent SHG behavior of the metasurface, we measured the SH power emitted into each diffraction order as a function of the FW using a fixed peak intensity of 0.33 GW/cm^2^, for either horizontal (Figure 5a) or vertical (Figure 5b) FW polarization. We found that the emission peaks at about 830 nm with a ∼50 nm spectral width of the metasurface resonance. Such spectral response reflects the major role played by the MD resonance in the nonlinear process, as also confirmed by the numerical simulations in Figure 5c and d. Both experiment and simulations confirm the presence of diffraction orders with polarization orthogonal to the pump polarization, which are up to 4 times weaker than the co-polarized ones and resonant to longer wavelengths. This behavior is corroborated by the polarization-resolved polar plots of the SH emission collected for the two orthogonal pump polarization states (see Figure 5e–h), which indicate that the diffraction orders aligned with the pump are co-polarized with it, while the orthogonal ones are cross-polarized. It is important to stress that the reported nonlinear diffraction sensibly differs from the linear diffraction at the SH wavelength, where the impinging linearly polarized light is diffracted mainly into the orders lying along the polarization direction and preserving the polarization state for all orders. In the SHG process, the polarization switching between different diffraction orders is ascribed to the unevenly distributed radial emission of the SH pattern of the individual meta-atoms, which originates from the interference between the nonlinearly excited MQ and the ED (see SI for details). The effective beaming of the in-plane field components of this radial mode into the respective diffraction orders endows the metasurface with its peculiar polarization and spectral selectivity. This adds another degree of freedom for controlling the SHG properties of our metasurface by engineering the emission of the individual meta-atom.

**Figure 5:**
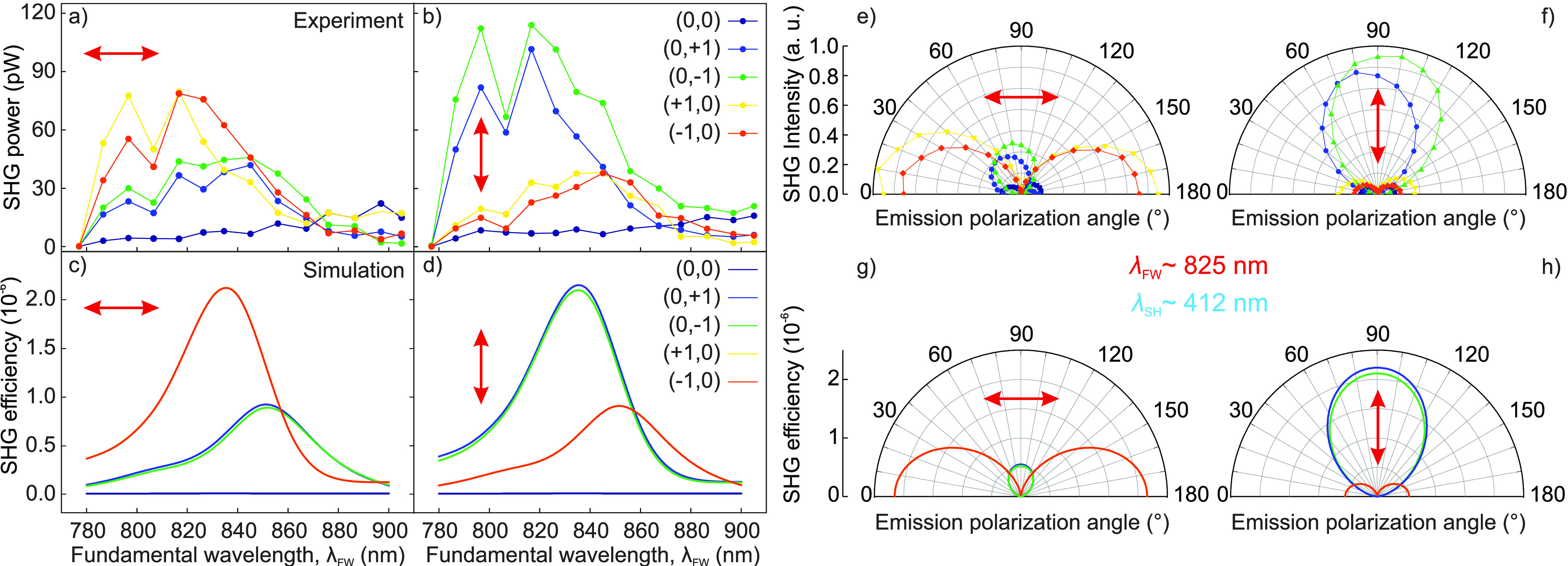
Wavelength-dependent SHG and polarization-resolved emission of the individual diffraction orders. (a, b) Measured SH power of each diffraction order as a function of the FW for horizontal (a) and vertical (b) pump polarization. (c, d) Simulated SHG efficiency of each diffraction order as a function of the FW for the same configurations as in (a) and (b). (e, f) Experimental polar plots showing the polarization state of the various diffraction orders for horizontal (e) and vertical (f) pump polarization. The FW employed to acquire the polar plots and the corresponding SH are ∼825 and ∼412 nm, respectively. (g, h) Simulated polar plots corresponding to (e) and (f).

To conclude, we have designed and realized a LiNbO_3_ nonlinear metasurface operating in the VIS range, featuring SHG performances in line with those of similar platforms^24^ operating in the NIR (nonlinear coefficient γ ≈ 3 × 10^–11^ W^–1^). In particular, thanks to the *z*-cut material and the metasurface periodicity, we observed a metasurface emission that is 10 times higher than that of the bare LiNbO_3_ film. This emission is directed predominantly toward the diffraction orders, while the (0,0) order is further suppressed by a factor of 10 in the metasurface. Therefore, our metasurface allows diffracting the SHG to the first diffraction orders modes with a signal 2 orders of magnitude stronger than the zeroth order, resulting in a ∼20 dB extinction ratio. Importantly, we report an intriguing polarization behavior of the emitted SH, which is redirected by the metasurface preferentially in the diffraction orders that lie along the pump polarization. These diffraction orders are co-polarized with the pump polarization, whereas the ones lying in the direction orthogonal to it are cross-polarized. At resonance, the extinction ratio between the co- and cross-polarized diffraction orders is about 4 (∼6 dB).

The possibility of efficient SH emission in the VIS range together with a polarization-controlled diffraction pattern make *z*-cut LiNbO_3_ metasurfaces promising tools for nonlinear holography with polarization encoding, having potential applications, for example, in anti-counterfeiting protection. Furthermore, the possibility of rerouting light with polarization selection could find further applications in free-space optical communications, such as Li-Fi.

## Supporting Information Available

Experimental setup, fabrication of the metasurface, effect of the sample geometry, SHG conversion efficiency and nonlinear parameter, numerical simulations of the linear response, numerical simulations of the SHG, multipolar analysis of the SH response (PDF)
