# Supplementary material for: Steering and Encoding the Polarization of the Second Harmonic in the Visible with a Monolithic LiNbO3 Metasurface
Source: ACS Photonics. 2021 Feb 19;8(3):731–7. doi: 10.1021/acsphotonics.1c00026 (PMC8029498; doi:10.1021/acsphotonics.1c00026)
Supplement: Supplementary file 1 — ph1c00026_si_001.pdf [file ph1c00026_si_001.pdf]

Supporting information to: Steering and Encoding the Polarization of Second Harmonic in the Visible with a Monolithic $LiNbO_3$ Metasurface

Luca Carletti,[†,‖] Attilio Zilli,[‡,‖] Fabio Moia,[¶] Andrea Toma,[¶] Marco Finazzi,[‡] Costantino De Angelis,[†] Dragomir N. Neshev,[*,§] and Michele Celebrano[*,‡]

†*Department of Information Engineering, University of Brescia, Via Branze 38, Brescia 25123, Italy*

‡*Politecnico di Milano, Physics Department, Piazza Leonardo da Vinci 32, 20133 Milano, Italy*

¶*Istituto Italiano di Tecnologia, Via Morego 30, 16163 Genova, Italy*

§*Australian National University, Nonlinear Physics Centre, 60 Mills Rd, Acton ACT 2601, Australia*

‖*These authors contributed in equal measure to this work*

E-mail: dragomir.neshev@anu.edu.au; michele.celebrano@polimi.it

S.I Experimental set-up

We employed the home-made nonlinear microscope depicted in Figure S1. A tunable (680 nm to 1080 nm) mode-locked Ti:Sapphire laser (Coherent, Chameleon Ultra II) provides 140 fs pulses with a repetition rate of 80 MHz. The laser light at the fundamental wavelength (FW) is loosely focused on the sample from the quartz substrate side via an achromatic doublet of $f = 60$ mm focal length. The resulting excitation spot is 15 µm in diameter. The second-harmonic (SH) emission is collected through a 0.85 NA air microscope objective (Nikon, CFI Plan Fluor 60XC), and chromatically filtered by two colored glass filters (FGB39 Schott-glass, Thorlabs) before detection. In order to investigate the directional properties of the emission, we imaged the back focal plane (BFP) of the objective. To do so, we inserted a Bertrand lens (BL, $f = 500$ mm achromat, Thorlabs) between the objective and the lens focusing onto our detector ($f = 500$ mm achromat, Thorlabs): a back-illuminated charge-coupled device (CCD) camera (Andor, iKon-M DU934P-BV). The lenses are placed in a $4f$ arrangement, therefore the BL focuses on an intermediate image plane, while the lens in front of the CCD re-collimates the beam. In this way, we reproduce a 1:1 telescope that images the BFP of the objective onto the CCD. Alternatively, for wavelength-resolved measurements, the collimated objective output is focused onto the entrance slit of a an imaging spectrometer (Andor, Shamrock SR-303i) equipped with a 150 grooves/mm ruled diffraction grating. The linear polarization of the exciting beam at 825 nm is rotated by a half-wave retarder (Thorlabs, WPH05M-808). Finally, the analysis of the emitted polarization is performed combining an achromatic half-wave retarder (Thorlabs, AHWP05M-600) with a Glan-Taylor polarizer (Thorlabs, GT10-A) inserted in the collimated portion of the detection path (see dashed area in Figure S1). For simplicity, here we omitted few optical elements (*e. g.* mirrors) which we account for when estimating the nonlinear conversion efficiency reported in Figure 4 in the paper and in Section S.IV.

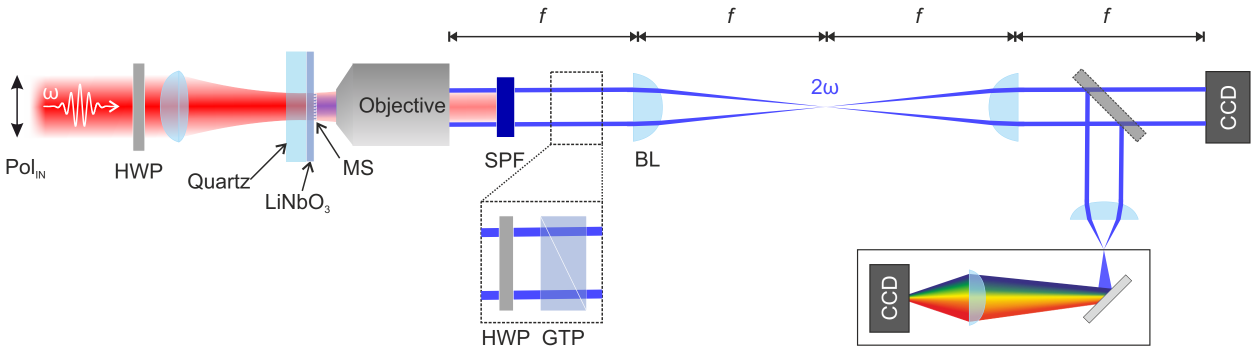

Figure S1: Simplified diagram of the experimental set-up. Acronym key: HWP = half-wave plate; MS = metasurface; SPF = short-pass colored glass filters; BL = Bertrand lens; GTP = Glan-Taylor polarizer; CCD = charge-coupled device camera. $f = 500$ mm.

S.II Fabrication of the metasurface

The metasurface was realized by nanostructuring a commercially available (NanoLN—Jinan Jingzheng Electronics Co.) z-cut $LiNbO_3$ film of 5 µm thickness grown on a transparent quartz substrate, as illustrated in Figure S2. Focused ion beam (FIB) milling was performed in a FEI - Dual Beam Helios Nanolab 650 machine. In this process, Ga^+ ions are emitted with a current of 200 pA and accelerated by a voltage of 30 keV. The overall ion dose was optimized to achieve a patterning depth of around 420 nm. Prior to ion milling, a 200 nm-thick Cr layer was deposited by radio frequency magnetron sputtering. The aforementioned sacrificial mask avoids charging effects during the FIB machining and promotes an optimal control over the nanostructure height, thus preventing size inhomogeneity across the manufactured surface. Concurrently, the Cr film acts as a sacrificial layer to prevent the implantation of Ga^+ ions and reduce the formation of defects inside the $LiNbO_3$ pillars.

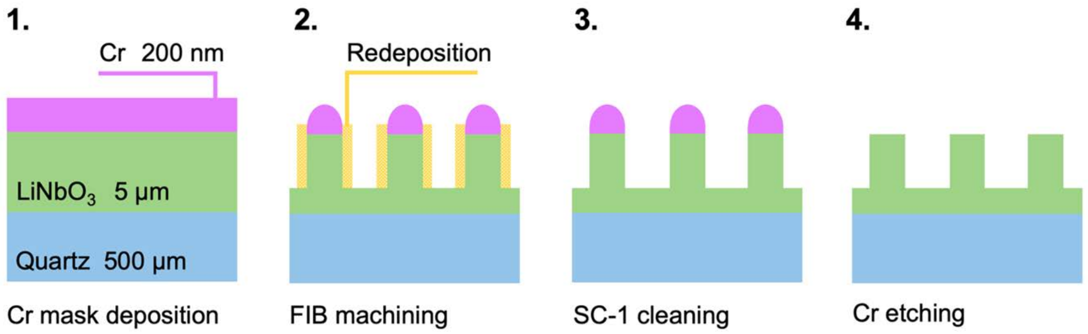

Figure S2: Flow-chart depicting the main steps of the metasurface fabrication.

The material removal that occurs during the ion milling results in re-deposition of $LiNbO_3$ near the impact zone (see Figure S2 panel 2)[S1]. This effect reduces the effective sputtering yield but concurs to the formation of a “self-protective” layer against additional ion implantation. In light of that, a chemical cleaning step based on SC-1 solution (70 % H_2O, 20 % H_2O_2, 10 % NH_4OH) was introduced to remove the etched and redeposited materials from the nanostructure side walls. Finally, the Cr hemispherical cap was dissolved in standard etchant solution (Chrome etch 18 - micro resist technology GmbH).

The complete collision cascade inside the $LiNbO_3$ surface has been evaluated recurring to Monte Carlo simulations (TRIM code)[S2,S3]. A detailed view of the Ga^+ trajectories into the target is reported in Figure S3a, where the lateral straggling after 4000 ions bombardment impinging at the position (X = 0, Y=0, Z=0) is depicted. As highlighted by the lateral distribution of collision events in Figure S3b, the region affected by ion milling is limited to the first nanometers, being almost negligible for lateral ranges exceeding 10 nm

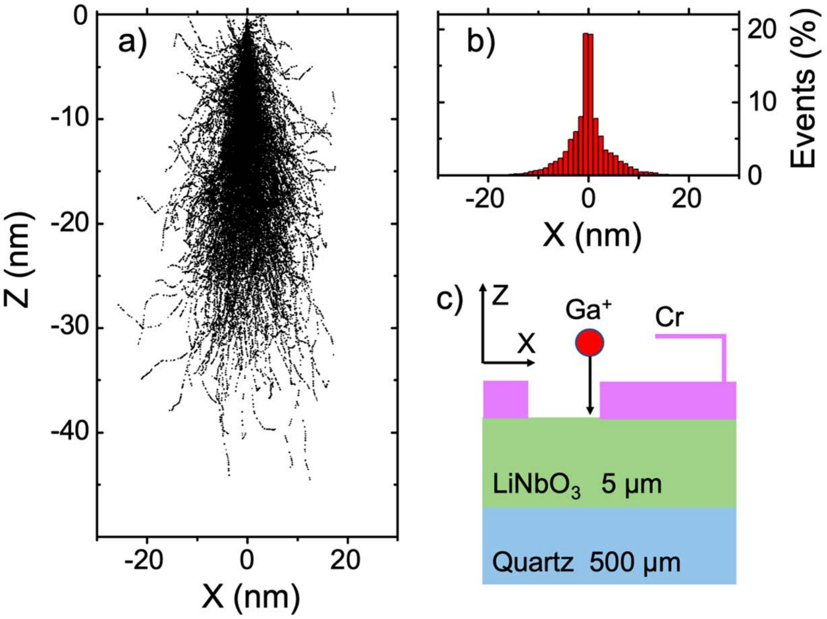

Figure S3: (a) Ga^+ trajectories within the $LiNbO_3$ film. (b) Lateral distribution of collision events spreading from the impact site. (c) Schematic representation of the FIB machining geometry adopted for TRIM calculation.

S.III Effect of the sample geometry

To finely tune the resonance near the FW of 830 nm, we realized 9 samples featuring variable geometrical parameters around the nominally optimum geometry. The experimentally acquired transmission spectra for the whole set of samples are shown in Figure S4a–c. The transmission spectra were obtained by illuminating the sample with an incoherent tungsten white light. Two main resonant features occur at about 680 nm and 790 nm, with relative weights that mostly depend on the radius R. Specifically, the first one dominates for the smallest value of $R = 150$ nm (panels a and d) whereas the second one is most intense for the largest value of $R = 200$ nm (panels c and f). A comparison between the experimentally acquired transmission spectra to the simulated ones in Figure S4d–f demonstrates the high level of accuracy attained by the FIB milling. Based on the multipolar analysis shown in Figure 2a of the Letter, the long wavelength resonance corresponds to a magnetic dipole and the short wavelength resonance corresponds to an electric dipole. The numerical simulations of the linear response Figure S5b,d,f display similar trends, including the infrared transmission dip red-shifting from 690 nm to 780 nm when R increases from 150 nm to 200 nm, as well as the increased transmission for smaller values of P.

The experimental second-harmonic generation (SHG) performance of the same 9 metasurfaces is illustrated in Figure S5, where 4 histograms report the intensity emitted in each diffraction order. The same polarization behavior described in the Letter is observed for all

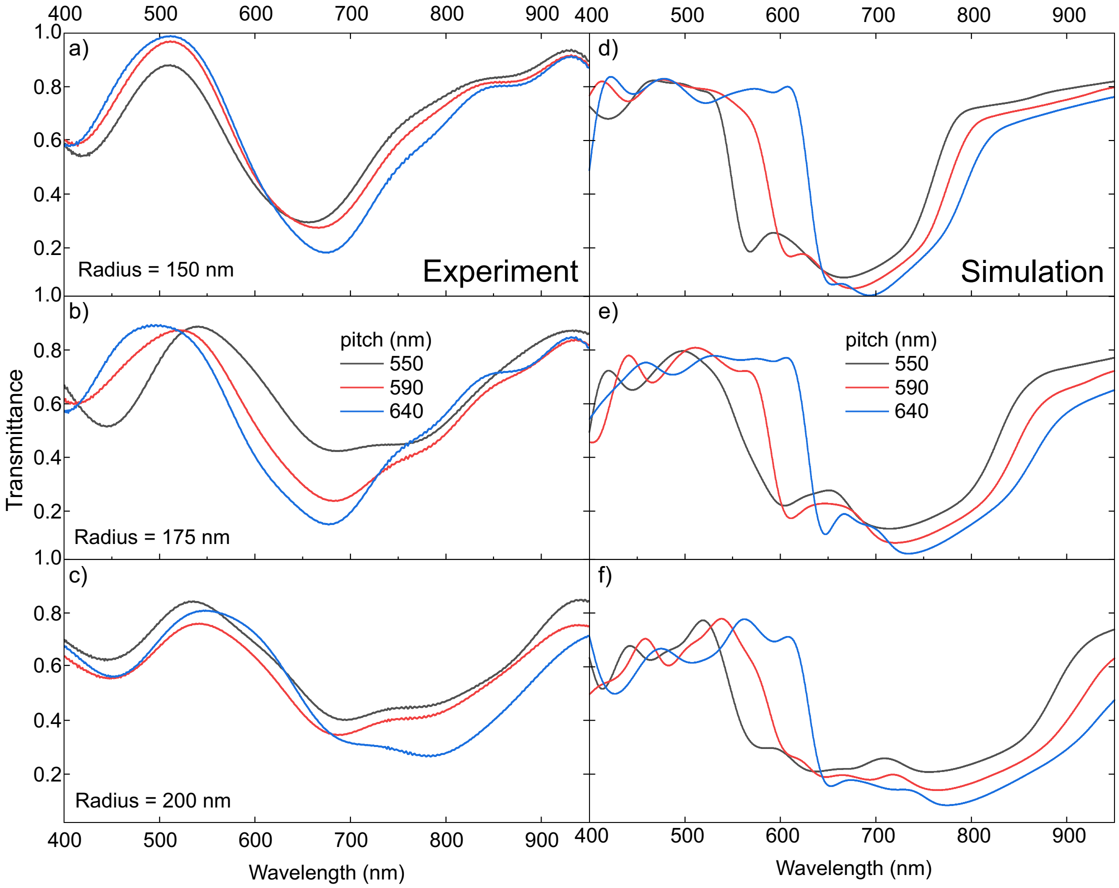

Figure S4: Linear transmission spectra of metasurfaces with varying radius and pitch. a–c) Measured transmission spectra for metasurfaces featuring nanopillars with radius $R = 150\,\mathrm{nm}$ (a), $175\,\mathrm{nm}$ (b) and $200\,\mathrm{nm}$ (c). The metasurface pitch is $550\,\mathrm{nm}$ (grey), $590\,\mathrm{nm}$ (red) and $640\,\mathrm{nm}$ (blue). d–f) Simulated transmission spectra of metasurfaces with the geometry of (a–c).

metasurfaces, namely the orders along a given direction are best excited by a pump polarized along that same direction. The most efficient metasurface is the one with $R = 175\,\mathrm{nm}$ and $P = 590\,\mathrm{nm}$, which is the one studied extensively throughout the Letter. Notably, the orders $(0, \pm 1)$ are always more intense than the $(\pm 1, 0)$ ones, suggesting either some systematic anisotropy induced in the sample by the fabrication process (*e. g.* along the fast scanning axis of the milling ion beam), or otherwise some small misalignment of the experimental set-up (*e. g.* some tilt in the exciting beam).

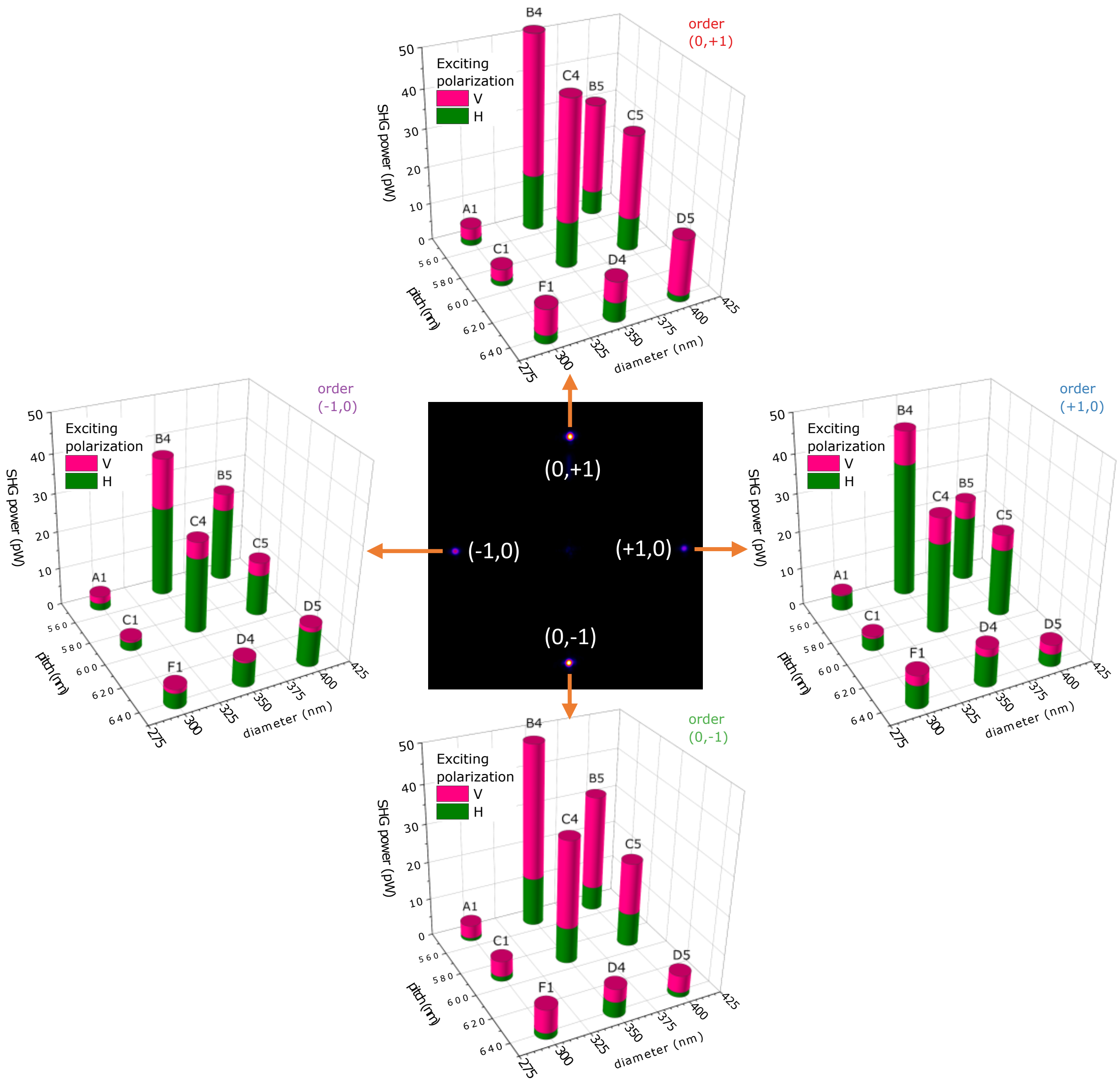

Figure S5: SH emission of gratings of metasurfaces with varying radius and pitch, for the two orthogonal polarization states of the pump.

S.IV SH conversion efficiency and nonlinear parameter

The SH conversion efficiency η of the metasurface is defined as $\eta \equiv P^{\text{SH}}_{\text{avg}}/P^{\text{FW}}_{\text{avg}}$ where $P^{\text{SH}}_{\text{avg}}$ and $P^{\text{FF}}_{\text{avg}}$ are the average powers of the SH emission and of the excitation at the FW powers. The nonlinear coefficient γ is defined in terms of the corresponding peak powers as $\gamma \equiv P^{\text{SH}}_{\text{pk}}/(P^{\text{FW}}_{\text{pk}})^2$, which makes it independent from the characteristics (*i. e.* fluence, pulse duration and repetition rate) of the laser source. Thus the relation $\eta = \gamma P^{\text{FW}}_{\text{pk}}$ holds between these two performance metrics.

Let us describe the procedure that we employed to determine η and γ in experiments. We measured $P^{\text{FW}}_{\text{avg}}$ with a photodiode power meter (Thorlabs, S122C) just before the sample, that is, on the back side of the quartz substrate. Thus, the power impinging on the metasurface is obtained by taking into account the reflection losses by the air–quartz ($n_{\text{quartz}} = 1.45$) interface of reflectance $R = 0.966$ and of the quartz–$LiNbO_3$ ($n_{\text{LiNbO}_3} = 2.25$) interface of reflectance $R = 0.953$. We measured $P^{\text{SH}}_{\text{avg}}$ using a CCD camera (Andor, iKon-M DU934P-BV) by summing the pixel values over the spot corresponding to each diffraction order, after subtraction of a dark frame. Such integrated number of counts, N_{c}, is proportional to the number of photoexcited electrons, N_{e}, via the sensitivity of the camera: $N_{\text{c}} = N_{\text{c}}/\varsigma$. We estimated $\varsigma = 3.9\,\text{ele/cnt}$ as the ratio of the electron well depth specified by the manufacturer to the single pixel saturation counts measured with the experimental acquisition settings. N_{e} is in turn proportional to the number N_{ph} of photons impinging on the pixel: $N_{\text{e}} = \text{QE} \times N_{\text{ph}}$, where $\text{QE} = 0.613$ is the quantum efficiency of the sensor at λ_{SH}. The corresponding radiant flux on the sensor is obtained by dividing the SH energy, $E_{\text{SH}} = N_{\text{ph}} \times hc/\lambda_{\text{SH}}$, by the exposure time of the frame. The power emitted by the sample is eventually deduced by dividing such power on the sensor by the transmittance at λ_{SH} of all the optical elements in the detection path, $T_{\lambda_{\text{SH}}}$. By considering the objective and filters transmittance, the optical interfaces and the mirrors reflectance, we arrive to a transmittance $T_{\lambda_{\text{SH}}} = 0.05$. The peak powers P_{pk} corresponding to the measured $P^{\text{SH}}_{\text{avg}}$ and $P^{\text{FW}}_{\text{avg}}$ are calculated as $P_{\text{pk}} = P_{\text{avg}}/(\tau\varpi)$ where $\tau = 140\,\text{fs}$ is the pulse duration and $\varpi = 80\,\text{MHz}$ is the pulse repetition rate.

S.V Numerical simulations of the linear response

The optical response of the $LiNbO_3$ metasurfaces is calculated using the commercial software COMSOL, that implements the finite element method. The geometry of the simulations is restricted to one unit cell of the metasurface and the excitation is a plane wave that is incident on the metasurface from the $LiNbO_3$ side. We account for the dispersion and birefringence of the refractive index of $LiNbO_3$ using the Sellmeier model[S4]

$$n_o^2(\lambda) = 1 + \frac{2.6734\lambda^2}{\lambda^2 - 0.01764} + \frac{1.2290\lambda^2}{\lambda^2 - 0.05914} + \frac{12.614\lambda^2}{\lambda^2 - 474.60} \quad \text{(S1-o)}$$

$$n_e^2(\lambda) = 1 + \frac{2.9804\lambda^2}{\lambda^2 - 0.02047} + \frac{0.5981\lambda^2}{\lambda^2 - 0.0666} + \frac{8.9543\lambda^2}{\lambda^2 - 416.08} \quad \text{(S1-e)}$$

where the wavelength λ is expressed in µm and the subscripts o and e stand for the ordinary and extraordinary optical axis respectively. We consider z-cut $LiNbO_3$ wafer (*i. e.* with the extraordinary axis parallel to the z-axis) as in our commercially-available substrate. The average intensity enhancement along the z-axis component obtained at the pump wavelength that is shown in Figure 3a of the Letter is defined as

$$\mathrm{FE_{avg}} = \frac{1}{V_\mathrm{d}} \int \left| \frac{E_z}{E_0} \right|^2 \mathrm{d}V \quad \text{(S2)}$$

where the integral is limited to the nanodisk volume, V_d, E_z is the z-axis component of the electric field, and E_0 is the amplitude of the incident electric field.

S.VI Numerical simulations of the SHG

The SHG process is reproduced using two cascaded simulations. In the first one, the optical response of the metasurface at the FW is computed. The electric field at the FW, E, induces a nonlinear polarization $\mathbf{P}^{\text{SH}}$ oscillating at the SH frequency in the $LiNbO_3$, whose second-order optical properties are described by the reduced nonlinear tensor d with elements $d_{22} = 2.1\,\mathrm{pm/V}$, $d_{31} = -4.3\,\mathrm{pm/V}$ and $d_{33} = -27\,\mathrm{pm/V}$.[S5] The components of $\mathbf{P}^{\text{SH}}$ thus are

$$\begin{aligned} \mathbf{P}_x^{\text{SH}} &= \varepsilon_0 \left(2d_{31}E_xE_z - 2d_{22}E_xE_y\right) \\ \mathbf{P}_y^{\text{SH}} &= \varepsilon_0 \left(-d_{22}E_x^2 + d_{22}E_y^2 + 2d_{31}E_yE_z\right) \\ \mathbf{P}_z^{\text{SH}} &= \varepsilon_0 \left[d_{31}(E_x^2 + E_y^2) + d_{33}E_z^2\right] \end{aligned} \quad \text{(S3)}$$

$\mathbf{P}^{\text{SH}}$ given by Eq. (S3) is the source term in the second simulation performed at λ_{SH}.

Figure S6 shows that the largest polarization component is $\mathbf{P}_z^{\text{SH}}$ since d_{33} is about one order

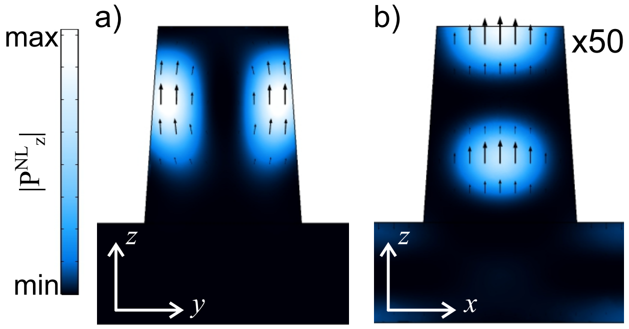

Figure S6: Nonlinear polarization in one meta-atom of the metasurface in the yz- (a) and zx-plane (b) for a y-polarized pump. $|\mathbf{P}_z^{\mathrm{SH}}|$ is represented in a false color scale while the arrow field represents $\mathbf{P}^{\mathrm{SH}}$. The geometrical parameters of the metasurface are $R = 175\,\mathrm{nm}$ and $P = 590\,\mathrm{nm}$. The scale in (b) is multiplied by a factor 50.

of magnitude larger than d_{31}, which determines the secondary terms of the SH polarization. Therefore, $\mathbf{P}^{\mathrm{SH}}$ lies in the xz plane for an x-polarized pump and in the yz plane for a y-polarized pump, as it can be noticed by comparing Figure S6a with Figure S6b. From such behaviour of the nonlinear polarization we can thus infer that the leading term of the SH emission will be co-polarized with the pump beam. These observations are in agreement with the observation of stronger co-polarized component of the SH and of the equal conversion efficiency observed for both pump polarization.

Let us now describe the procedure to determine η and γ in the numerical simulations that are compared to the experimental results. Note that we solve the wave equation in the frequency domain, which means that the temporal structure of the pulsed excitation is not taken into account. Given the instantaneous nature of the nonlinear phenomena that we consider, the simulated fields and powers must therefore be interpreted as instantaneous peak values. In light of this, we evaluate the nonlinear parameter γ considering the overall emission directed towards the air side of the metasurface. We estimate, numerically, $\gamma \approx 3.7 \times 10^{-6}\,\mathrm{W}^{-1}$. To estimate the SHG parameters of the fabricated metasurfaces we fix the intensity of the pump to be equal to the experimental one. Using a peak intensity of $0.5\,\mathrm{GW/cm}^2$, the peak power in each cell is $1.74\,\mathrm{W}$. With this value, the numerically estimated conversion efficiency is $\eta = \gamma \times P_{\mathrm{pk}}^{\mathrm{FW}} \approx 5 \times 10^{-6}$. Discrepancies between the numerical and experimental results might be due to different factors such as the uncertainties in the estimation of the input intensity and of the optical transmittance of the detection path as well as the different excitation configurations (*i.e.* a focused beam in the experiment and a plane wave in the simulations). Also side wall roughness and small fabrication imperfections may concur to decrease the experimental efficiency.

S.VII Multipolar analysis of the SH response

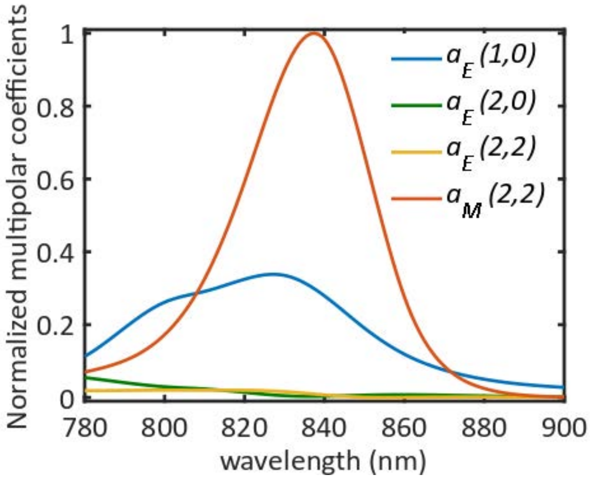

Figure S7: Multipolar contributions to the SHG response. The electric dipole, $a_{\mathrm{E}}(1,0)$, and magnetic quadrupole, $a_{\mathrm{M}}(2,2)$, contributions are more than one order of magnitude larger than the electric quadrupole contributions, $a_{\mathrm{E}}(2,0)$ and $a_{\mathrm{E}}(2,2)$.

We decomposed the electric field at the SH frequency in the nanopillars in spherical harmonics, obtaining that the main multipolar contributions are the electric dipole $a_{\mathrm{E}}(1,0)$ and magnetic quadrupole $a_{\mathrm{M}}(2,2)$ (see Figure S7). The spherical vector harmonics, in spherical coordinate system (r,θ,ϕ) with origin at the center of the nanopillars, that are associated with these multipoles in the limit $r \longrightarrow \infty$ are[S6]

$$a_{\mathrm{E}}(1,0) \longrightarrow \mathbf{N}_{e10} = -\sin\theta \frac{ie^{i\rho}}{\rho}\hat{e}_\theta \tag{S4}$$

$$a_{\mathrm{M}}(2,2) \longrightarrow \mathbf{M}_{o22} = 6\sin\theta\cos(2\phi)\frac{ie^{i\rho}}{\rho}\hat{e}_\theta - 6\sin\theta\cos\theta\sin(2\phi)\frac{ie^{i\rho}}{\rho}\hat{e}_\phi \tag{S5}$$

where $\rho = rk$ with k the wavevector. The scattered electric field can be obtained as the sum of all spherical multipoles. Considering only the main contributions from the electric dipole and magnetic quadrupole we can write

$$\mathbf{E} \sim -\frac{3}{2}a_{\mathrm{E}}(1,0)\mathbf{N}_{e01} - \frac{5}{6}a_{\mathrm{M}}(2,2)\mathbf{M}_{o22} \tag{S6}$$

Eq. (S6) should be evaluated using (θ,ϕ) angles that correspond to the observed diffraction orders. In detail, the zeroth order will be at (0°,0°), the $(\pm 1,0)$ orders will be at $(44.7°,0°)$ and $(44.7°,180°)$, the $(0,\pm 1)$ orders will be at $(44.7°,90°)$ and $(44.7°,270°)$. Let us focus on the first diffraction orders for which $\theta = 44.7°$. From Eq. (S4) and Eq. (S5) we can notice that the ϕ component of the electric field is determined only by the magnetic quadrupole

and it always cancels out for the allowed values of ϕ. On the other hand, the θ component of the electric field will be

$$\mathbf{E} \cdot \hat{e}_\theta = E_\theta \propto \sin\theta \left[\frac{3}{2} a_{\mathrm{E}}(1,0) - 5\cos(2\phi) a_{\mathrm{M}}(2,2)\right] \tag{S7}$$

As it can be observed, the contributions from the two multipoles add destructively or constructively as a function of ϕ. In particular, E_θ is periodic in ϕ with a periodicity of 180° and thus either the $(\pm 1, 0)$ or the $(0, \pm 1)$ diffraction orders will dominate emission depending on the phase of the multipolar coefficients (which is determined by the polarization of the pump beam). In addition, as neither the electric dipole nor the magnetic quadrupole radiate along the normal ($\theta = 0$) direction, the SH emitted along the zeroth diffraction order is negligible.

The multipolar analysis can motivate the position and polarization of the main SH diffraction orders that are observed in both numerical simulations and experiments. The intensity of the emitted field in the $(\pm 1, 0)$ and $(0, \pm 1)$ diffraction orders at the SH wavelength for an x-polarized pump beam as a function of the pump wavelength that is obtained from Eq. (S7) is shown in Figure S8. As it can be seen, the highest peak occurs for $\phi = 0$ (corresponding to the $(\pm 1, 0)$ diffraction orders) at wavelength of 830 nm. For $\phi = 90°$ (corresponding to the $(0, \pm 1)$ diffraction orders), we can observe a secondary SH peak at a wavelength of 845 nm. This stems from the dimmering of the electric dipole contribution at these wavelengths that reduces the interference effect between the two multipoles. These results are in striking agreement with the polarization-resolved spectra in Figure 5e–h of the Letter.

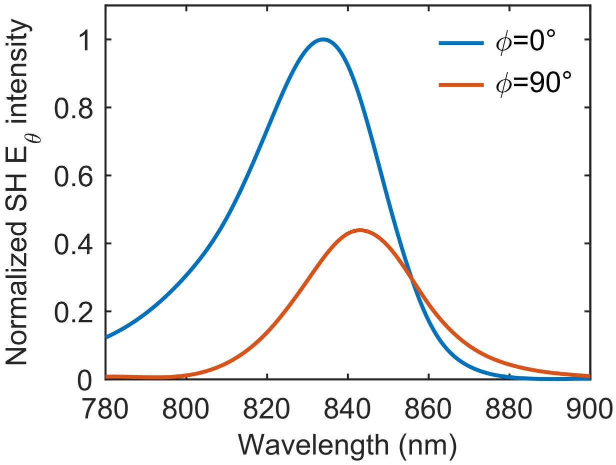

Figure S8: Intensity of the emitted field at the SH wavelength for an x-polarized pump beam as a function of the pump wavelength due to the electric dipole, $a_{\mathrm{E}}(1,0)$, and magnetic quadrupole, $a_{\mathrm{M}}(2,2)$, contributions. In this calculation $\theta = 44.7°$ and ϕ is either 0 or 90°.

References

(S1) Ulliac, G.; Calero, V.; Ndao, A.; Baida, F.; Bernal, M.-P. Argon plasma inductively coupled plasma reactive ion etching study for smooth sidewall thin film lithium niobate waveguide application. *Opt. Mater.* **2016**, *53*, 1–5.

(S2) Biersack, J.; Haggmark, L. A Monte Carlo computer program for the transport of energetic ions in amorphous targets. *Nuclear Instruments and Methods* **1980**, *174*, 257–269.

(S3) Ziegler, J. F.; Ziegler, M.; Biersack, J. SRIM – The stopping and range of ions in matter (2010). *Nuclear Instruments and Methods in Physics Research Section B: Beam Interactions with Materials and Atoms* **2010**, *268*, 1818–1823, 19th International Conference on Ion Beam Analysis.

(S4) Zelmon, D. E.; Small, L.; Jundt, D. Infrared corrected Sellmeier coefficients for congruently grown lithium niobate and 5 mol. *J. Opt. Soc. Am. B* **1997**, *14*, 3319–3322.

(S5) Simplified characterization of uniaxial and biaxial nonlinear optical crystals: a plea for standardization of nomenclature and conventions. *IEEE Journal of Quantum Electronics* **1992**, *28*, 2057–2074.

(S6) Bohren, C. F.; Huffman, D. R. *Absorption and Scattering of Light by Small Particles*; John Wiley & Sons, Ltd, 1998; Chapter 3, pp 57–81.
